# The impact of spontaneous intracranial hypotension on social life and health-related quality of life

**DOI:** 10.1007/s00415-022-11207-7

**Published:** 2022-06-14

**Authors:** Christopher Marvin Jesse, Levin Häni, Christian Fung, Christian Thomas Ulrich, Ralph T. Schär, Tomas Dobrocky, Eike Immo Piechowiak, Johannes Goldberg, Christoph Schankin, Harri Sintonen, Jürgen Beck, Andreas Raabe

**Affiliations:** 1 Department of Neurosurgery, Inselspital, Bern University Hospital, University of Bern, Freiburgstrasse 10, 3010 Bern, Switzerland; 2 Department of Neurosurgery, Medical Center – University of Freiburg, Freiburg, Germany; 3 Department of Neurosurgery, Lindenhofspital, Bern, Switzerland; 4 Institute of Diagnostic and Interventional Neuroradiology, Inselspital, Bern University Hospital, University of Bern, Bern, Switzerland; 5 Department of Neurology, Inselspital, Bern University Hospital, University of Bern, Bern, Switzerland; 6 Department of Public Health, University of Helsinki, Helsinki, Finland

**Keywords:** Spontaneous intracranial hypotension, Spinal cerebrospinal fluid leak, Orthostatic headache, Health-related quality of life

## Abstract

**Objective:**

Spontaneous intracranial hypotension (SIH), which is often caused by a spinal cerebrospinal fluid leak, is an important cause of disabling headaches. Many patients report devastating changes in their quality of life because of their symptoms. This study aimed to evaluate the impact of SIH on patients’ social/ working life and health-related quality of life (HRQoL).

**Methods:**

We included consecutive patients with proven SIH treated at our institution from January 2013 to May 2020. Patients were contacted and asked to complete the 15D questionnaire for the collection of HRQoL data and to provide additional information on their social life status.

**Results:**

Of 112 patients, 79 (70.5%) returned the questionnaire and were included in the analysis. Of those, 69 were treated surgically (87.3%), and 10 were managed non-operatively (12.7%). Twenty-five (31.6%) patients reported a severe impact on their partnership, 32 (41.5%) reported a moderate or severe impact on their social life. Forty (54.8%) patients reported sick leave for more than 3 months. The mean 15D score was 0.890 (± 0.114) and significantly impaired compared to an age- and sex-matched general population (*p* = 0.001), despite treatment. Patients with residual SIH-symptoms (36, 45.6%) had significantly impaired HRQoL compared to those without any residual symptoms (41, 51.9%) (*p* < 0.001).

**Conclusion:**

SIH had a notable impact on the patients’ social life and HRQoL. It caused long periods of incapacity for work, and is therefore, associated with high economic costs. Although all patients were appropriately treated, reduced HRQoL persisted after treatment, underlining the chronic character of this disease.

## Introduction

Spontaneous intracranial hypotension (SIH) is an important cause of incapacitating headache. It has an estimated incidence of 5/100,000 and is primarily caused by a cerebrospinal fluid (CSF) leak in the cervicothoracic part of the spine [1–4]. The hallmark symptom is orthostatic headache, but patients often report other symptoms such as orthostatic visual or vestibulocochlear manifestations [5]. Throughout the disease, the symptoms can change and present a more chronic character with fewer orthostatic components [6, 7]. This chronification of initial orthostatic symptoms is accompanied by changes in CSF dynamics [6].

Treatment of SIH consists of conservative measures like bed rest, oral caffeine and epidural blood patching [8], as well as surgical closure of the leak and endovascular embolization [9, 10]. Longer duration of preoperative symptoms is associated with a higher risk of residual complaints after surgical treatment. Thus, an early diagnostic work-up and definitive closure of the leak in patients is advisable [11]. Patients may not only experience residual symptoms but also rebound headache, which occurs in about a quarter of patients following surgical and conservative treatment of SIH [12]. The latter may also lead to a significant impairment of quality of life in many cases.

The aim of our study was to assess the influence of SIH on the social life and working capacity, as well as health-related quality of life (HRQoL) of patients who had been treated for SIH. We hypothesized that SIH has a significant impact on their daily and social life. Analyzing the outcome of this condition is crucial for raising awareness among clinicians and health insurance companies.

## Methods

### Study design

We conducted a retrospective, observational case–control study. Patients diagnosed with SIH due to spinal CSF leakage were surveyed using a specifically designed questionnaire including the 15D questionnaire. Approval from the local ethics committee of the canton of Bern, Switzerland, was obtained for this study (2020–00645). Only patients who had given approved general consent with permission for the use of their health-related data were included.

### Patient selection and data collection

Patients with neuroradiologically proven SIH due to a spinal CSF leak treated at our institution between January 2013 and May 2020 were included in this study. Patients treated for secondary CSF leakage, e.g., following previous lumbar puncture, epidural anesthesia or spinal surgery, were excluded. The questionnaire was sent by mail to all patients who met the inclusion criteria. If there was no response, we tried to contact the patient by phone and asked her or him to answer a digital version of the questionnaire. After three failed attempts, the data were considered missing and were excluded from the analysis.

The patient-specific data as well as data regarding the surgery, radiologic findings, and perioperative course have been collected prospectively in our SIH database and were retrospectively analyzed.

### SIH standard of care regarding diagnostic work-up and treatment

All patients received a standardized stepwise diagnostic work-up proceeding from non-invasive (e.g. MRI, optic nerve sheath ultrasound) to more invasive techniques (e.g. dynamic myelography) depending on the level of suspicion of a spinal CSF leak. Patients with intracranial signs of hypotension, utilizing the SIH-score [13], and no spinal longitudinal epidural CSF collections (SLEC negative), lateral decubitus digital subtraction myelography and postmyleography CT were used to look for a CSF-venous fistula. Our preoperative diagnostic work-up has been described previously [14–16].

Conservative treatment consisted of bed rest, oral caffeine and/or non-targeted epidural lumbar blood patching. If a spinal CSF leak was found and clearly localized and conservative treatment failed to provide long-term relief, surgical closure of the leak was performed [9, 17]. Two months after treatment, we performed an additional MRI-scan of the spine and head and in cases with suspicion of ongoing CSF leak, we repeated the diagnostic work-up.

### Study-specific questionnaire

A study-specific questionnaire with open, numeric and multiple-choice questions was designed. The questions covered the symptoms and their duration, previous treatment and post-treatment changes, success of treatment and the effects on the patient’s social life and work capacity before and after treatment. Parts of the questionnaire and the results regarding the surgically treated patients as well as the implications have been presented in a previous publication [11].

### Health-related quality of life (HRQoL) by 15D

In addition to the study-specific questionnaire, patients were asked to complete the self-administered, generic 15D questionnaire to evaluate their status at the time point of contact. The 15D is a health-related quality of life (HRQoL) questionnaire containing 5 levels for each of 15 dimensions, namely: mobility, vision, hearing, breathing, sleeping, eating, speech, excretion, usual activities, mental function, discomfort and symptoms, depression, distress, vitality, and sexual activity. Each dimension was converted to a 0–1 scale (dimension level values) by applying the Finnish multi-attribute utility weights. The single index score (15D score), which is calculated from these 15 dimensions, represents the overall HRQoL and ranges from 0 (death) to 1 (totally healthy) [18]. The minimum clinically important change or difference (MIC) was defined as ± 0.015 [19].

The single index 15D score as well as the profile of the different dimensions were compared with that of a representative age- and sex-standardized sample of the Finnish population [20]. The study-specific questionnaire and the 15D were available in German and French.

### Statistics

Descriptive data included calculation of the mean and standard deviation (SD). Matched samples were compared with Wilcoxon’s signed-rank test. Two-way comparisons between groups were made with the Mann–Whitney *U* test for continuous variables, and chi-squared or Fisher’s exact test for nominal variables. To test for the correlation of early treatment, we divided the patients in an early treated group (≤ 12 weeks) and late treated group (> 12 weeks). The mean 15D scores and dimension level values were compared between groups with an independent samples *t* test. Missing data for the 15D questionnaire were replaced according to the algorithm of the developer of the questionnaire [18]. We addressed missing values first by re-analyzing the source data or, second, if no value was retrievable, by pairwise deletion. Statistical analysis was performed using the statistical software SPSS (IBM, version 25). Statistical significance was defined as a *p* value less than 0.05.

## Results

### Patient characteristics

**Fig. 1:**
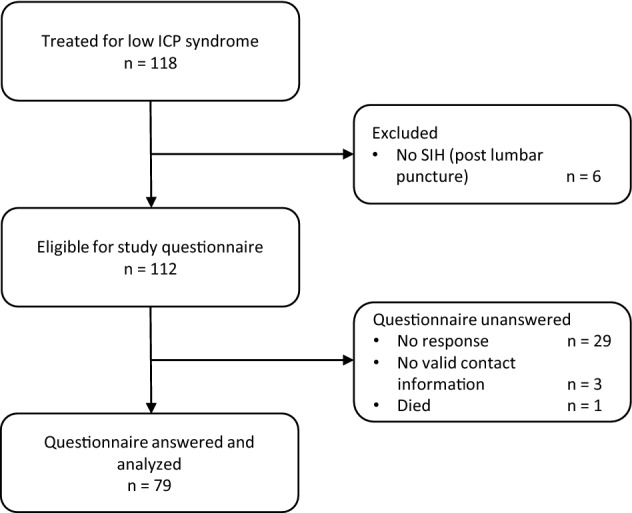
Flow diagram of patients included in the study

From January 2013 to May 2020, 118 patients with low ICP syndrome were treated in our department. Six patients with postdural puncture headache were excluded. The remaining 112 patients with true SIH were selected for study participation and were contacted to answer the questionnaire. Twenty-nine patients did not return the questionnaire, for three patients, there was no valid contact information and one 78-year-old female patient had died. Thus, a total of 79 patients (70.5%) returned the questionnaire and were included in the analysis (Fig. 1).

The mean age at return of the questionnaire was 47.7 (± 12.6) years and mean age at time of treatment was 45.7 (± 12.3) years. Fifty-three patients (67.1%) were female. Of the 79 patients, 69 had been treated surgically (87.3%) and 10 non-operatively (12.7%). Whereas the response rate was 80.2% (69 out of 86) in the group that had undergone surgery, the response rate in the non-surgical group was only 38.5% (10 out of 26). The treatment and outcome of the patients in the surgery group have been described in an earlier publication [11]. In the non-operative group, 80% (8 out of 10) of the patients had received an untargeted lumbar epidural blood patch. In 4 patients, the site of the leakage was in the cervical spine, in 1 at the cervicothoracic junction, in 59 in the thoracic region, in 7 at the thoracolumbar junction, and in 1 patient in the lumbar spine. For 7 patients, the exact site of the leak remained unknown.

### Symptoms and course

Whereas 67 (84.8%) of patients reported orthostatic headache in the first weeks after developing symptoms, 12 (15.2%) patients did not. However, 29 (36.7%) patients reported a non-orthostatic headache during this time. Fifty-seven patients (72.2%) described an evolution of the symptoms over the course of the disease.

After any kind of treatment, 41 (51.9%) patients reported a complete resolution of symptoms, 32 (40.5%) reported partial improvement, and 4 (5.1%) could not detect any improvement. The resolution of symptoms correlated with the time to treatment. The early treated group showed significant better improvement after treatment than the late treated group (18/24 patients (75%) versus 12/33 patients (36.4%) with complete resolution of symptoms; *p* = 0.011) Mean pre- and postoperative headache intensities on the numeric rating scale were 8.38 (± 1.8) and 1.50 (± 2.0), respectively (*p* < 0.001). Additionally, 65 patients (94.2% of those in the surgery group) declared that they would choose to undergo treatment again. There was no significant difference between the sexes in their symptom resolution after treatment (*p* = 0.81).

### Impact on social life

**Fig. 2:**
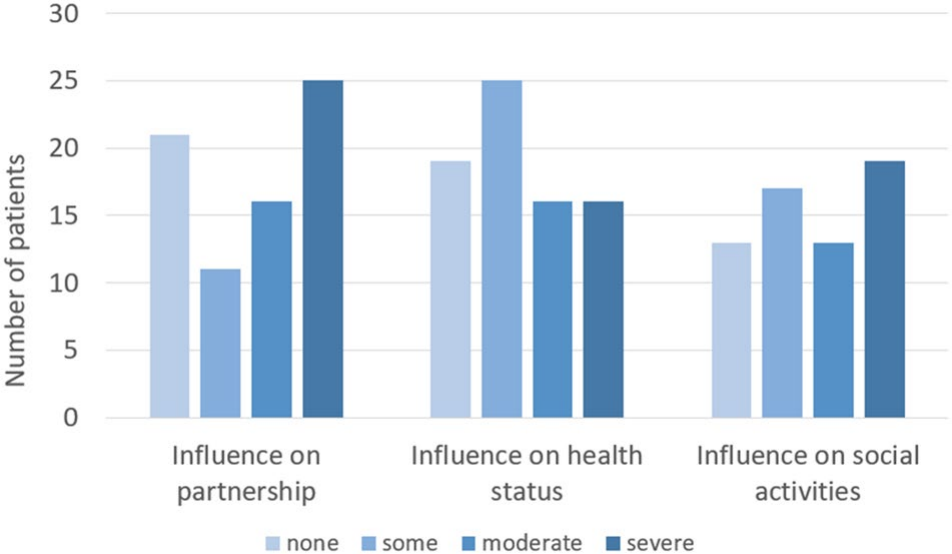
Impact of SIH on social life. Patients were asked about the influence of SIH on their relationship with their partner, current health status and social activities

Twenty-five (31.6%) patients reported a severe impact of the disease on their relationship with their partner, 16 (20.3%) a moderate impact, 11 (13.9%) some impact and only 21 (26.6%) reported no impact. Additionally, 5 (6.3%) patients stated that the disease had led to a divorce. Notably, 32 (41.5%) patients reported a continuing moderate or severe impact on their social life despite treatment at the time of the study, whereas only 30 (38%) of patient experienced no continuing impact. Furthermore, 16 (20.3%) of the patients said that the disease still had a severe impact on their current health status, and 16 (20.3%) and 25 (31.6%) patients still noticed a moderate or small impact on their current health status. Only 19 (24.1%) patients reported that the disease no longer had any impact. When asked whether the disease had changed their perception of the role of their health status, 62 (78.5%) answered that it had. When asked to rate their mean life satisfaction regarding their health status on a scale from zero to one hundred at present, the mean satisfaction score was 81 (± 21) (Fig. 2).

### Impact on work life

**Fig. 3:**
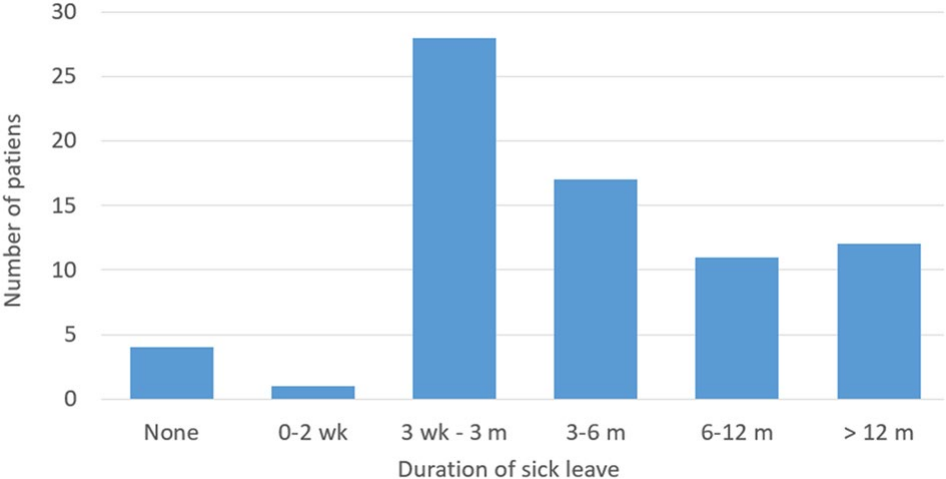
Duration of sick leave after onset of symptoms due to SIH (wk: weeks; m: months)

Mean total absence from work was 30 weeks (± 40.3 weeks). More than half (40/73, 54.8%) of the patients reported taking sick leave due to the disease for more than 3 months. Only 5 (6.3%) patients reported taking no sick leave or less than 2 weeks. The return-to-work rate 1 year after symptom onset was 83.6% (Fig. 3). While 53 (67.1%) patients were fully able to return to work, 19 (24.1%) were not. Moreover, 8 (10.1%) patients had to retire early due to the symptoms of SIH. Of the patients with complete symptom resolution, 86.8% (33/38) were able to return to the same work pattern as before the disease, whereas only 56.3% (18/32) of patients with residual symptoms did so (*p* = 0.03). Eleven (13.9%) patients had to change jobs due to the symptoms of the disease.

### Health-related quality of life (HRQoL)

**Fig. 4:**
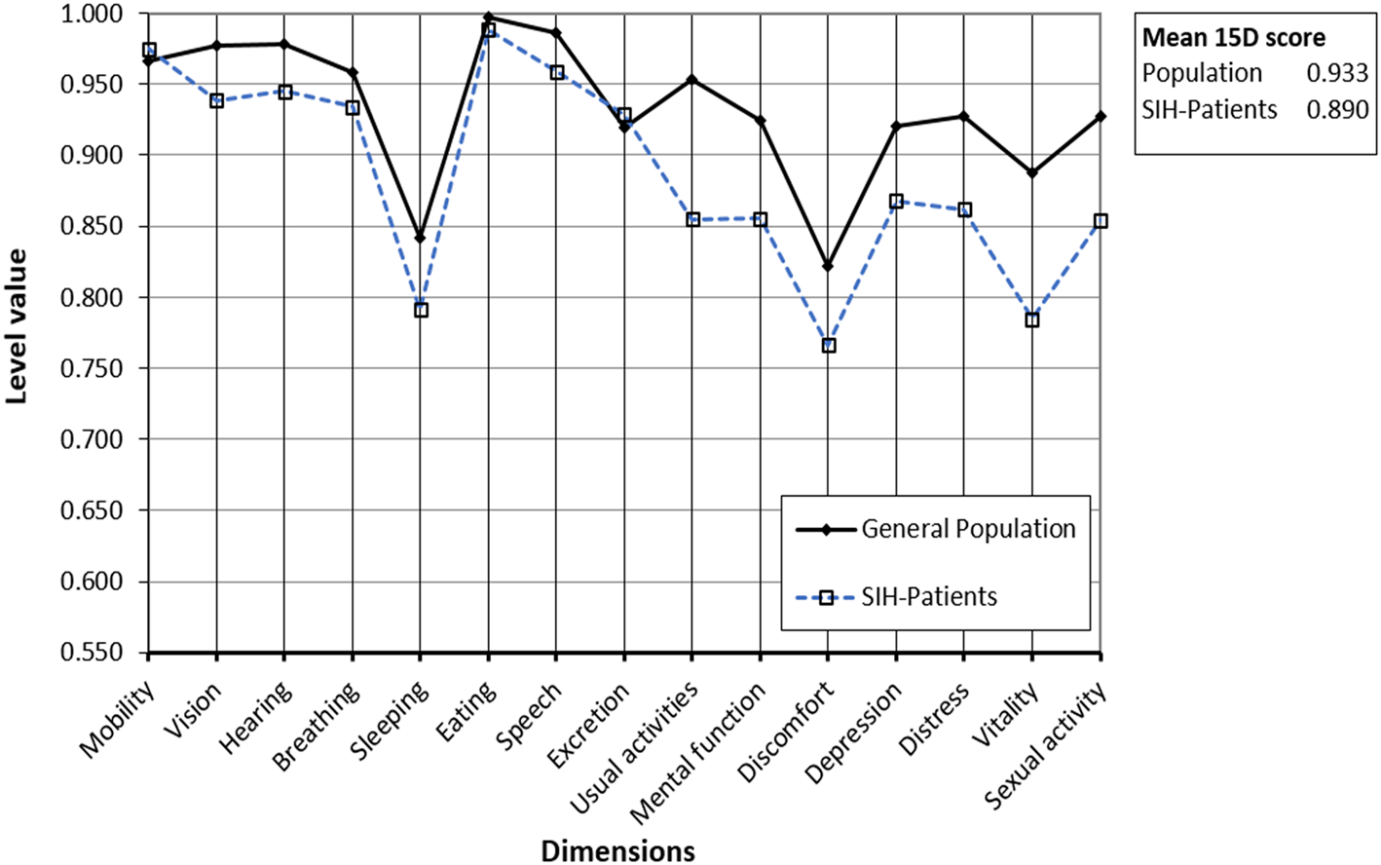
The mean 15D score and profile of SIH patients and those of an age- and sex-standardized sample of the general population. Dimensions and level of significance are shown in Table 1

The mean 15D score in the presented cohort was 0.890 (± 0.114). SIH patients had a significantly impaired mean 15D score compared to a sex- and age-standardized group (*n* = 1209) from the Finnish population (mean 15D score of 0.933; *p* = 0.001). Comparing the different dimensions of the 15D, the SIH group was significantly worse off on the dimensions of vision, hearing, speech, usual activities, mental function, depression, distress, vitality and sexual activity (Fig. 4, Table 1). With a mean difference of 0.043 in the 15D score between these two groups, the difference was clinically important.

**Table 1:** Comparison of the different dimensions of the 15D questionnaire between treated SIH patients and the general population. A positive difference indicates that the population score is better.

Dimension	Mean difference	Standard error difference	95% confidence interval	Significance
Mobility	− 0.008784	0.010825	− 0.030267	0.419
Vision	0.038156	0.014880	0.008542	0.012
Hearing	0.032544	0.013924	0.004849	0.022
Breathing	0.023633	0.016871	− 0.009907	0.165
Sleeping	0.050149	0.026044	− 0.001645	0.058
Eating	0.008953	0.008491	− 0.007950	0.295
Speech	0.026719	0.012758	0.001337	0.039
Excretion	− 0.008949	0.016305	− 0.041359	0.585
Usual activities	0.098424	0.027298	0.044092	0.001
Mental function	0.069358	0.022707	0.024189	0.003
Discomfort and symptoms	0.055385	0.030739	− 0.005790	0.075
Depression	0.052418	0.022272	0.008101	0.021
Distress	0.064914	0.020841	0.023452	0.003
Vitality	0.102335	0.027466	0.047682	0.000
Sexual activity	0.073122	0.027467	0.018493	0.009
15D score	0.043085	0.012922	0.017380	0.001

The 15D score in patients with residual symptoms after treatment was 0.838 (± 0.123) compared to 0.938 (± 0.082) in patients with complete symptom resolution, showing a clinically important difference between these two groups (*p* < 0.001). Additionally, of the 15 dimensions of the 15D questionnaire, 10 (moving, breathing, speech, usual activities, mental activities, discomfort and symptoms, depression, distress, vitality and sexual activity) were significantly impaired in patients whose symptoms had only partially resolved after treatment (*p* < 0.05). There was no significant difference between the surgically and non-surgically treated groups in the mean 15D score (*p* = 0.355). Women reported a lower level value on the dimension of breathing than men did (*p* = 0.03). There was no significant sex-specific difference in any of the other dimensions or for the 15D score.

## Discussion

Our findings suggest an important impact of SIH on the social and work life of patients. HRQoL is significantly and clinically importantly impaired in SIH patients compared to the general population. Despite treatment of the leak, there is a high rate of patients with residual symptoms.

### Impact on social life

Our patients reported long periods of sick leave due to the symptoms of SIH. More than half of the group was on sick leave for more than 3 months and nearly one in four patients were unable to return to work under the same job conditions as before onset of SIH. With an estimated incidence of 5/100,000 [1], this disease has a notable individual and socio-economic impact.

The ability of patients to return to work was associated with the extent of symptom resolution. The full resumption of work rate was about 30% lower in the group with persisting symptoms. In our previous study, the difference in the surgically treated patients was even higher. Of the patients with complete resolution of symptoms, 90.9% were able to return to working as they did before the disease, but only 56.7% of patients with persisting symptoms did so [11]. Taking this finding into account, together with the fact that symptoms and CSF dynamics change over the duration of the disease and tend to become chronic [6], it seems advisable to aspire to complete symptom relief with early treatment.

Comparing with other diseases or trauma, SIH patients had a lower rate of return to work after 1 year than those with surgically treated spinal tumors or spinal fractures (83.6% versus 90% for each) [21]. This might be because the symptoms of SIH patients are often misunderstood and there is myriad of symptoms especially in long lasting disease. Therefore, it takes a long time to reach the correct diagnosis [11].

### Impact on health-related quality of life (HRQoL)

HRQoL after treatment was impaired in our cohort of SIH patients. The mean difference of 0.043 of the 15D score between SIH patients and the general population is not only clinically important but stands for a *much worse* HRQoL [19]. SIH patients showed a similar mean decline in HRQoL to that seen in patients with other diseases, for example, inflammatory bowel disease [22].

Although all our SIH patients received appropriate treatment, the HRQoL remained impaired. Since the questionnaire was sent to the patients about 2 years after treatment, the deterioration of HRQoL underlines the chronic character of this disease even after treatment. A clinically important difference between patients with and without complete symptom resolution after treatment was seen in our group. Interestingly, patients with a complete symptom resolution have a similar HRQoL like the normal population (15D score: 0.938 compared to 0.933). Additionally, an earlier study of our patient group showed better outcome and symptom relief for early treated patients [11], indicating that early treatment leads to a higher HRQoL in the long run.

In a recent study, Chee et al. performed an online survey of SIH patients, and demonstrated a comparable impact on working capacity and HRQoL [23]. The authors reported that a quarter of patients had lost their job and 60% had had to alter their work duties due to SIH. Additionally, they showed a negative impact on quality of life as measured by the EQ-5D-5L questionnaire. The authors concluded that SIH is a highly disabling disorder affecting multiple domains, which is in line with our findings. However, treatment modality in their study population differed from ours: Whereas only 9.8% of patients in that study were treated surgically, we surgically addressed the leak in 87.3% of the patients. Even though comparisons of these study populations should be done cautiously and we do not know for all cases whether the leak was really closed after surgery, our patients reported a mean life satisfaction regarding their health status of 81 on a scale from 0 to 100, whereas the patients surveyed in the British study reported a mean EQ-5D-5L Visual Analogue Scale score (from 0 to 100) of only 36.4. That may indicate that there were still patients in their cohort with chronic symptoms, possibly due to delayed treatment or ongoing CSF leakage.

Interestingly, although 51.9% of patients reported a complete resolution of symptoms after treatment, only 24.1% stated that the disease has no impact on their current health status anymore and HrQoL remained reduced. We can only speculate why this is the case. Since many patients reported that the disease had changed their personal importance of health this could be an explanation. Another explanation might be that patients developed new symptoms after some time following treatment and they attributed these symptoms to SIH. Third, the development of other neurological or psychosomatic factors e.g. functional overlay or triggering of other types of headache might also play a role. Finally, the ongoing impact on their health status could be explained by the social and economic consequences of the disease. Since some patients had to retire, change their job or got divorced because of the disease this could lead to an ongoing impact on their health status, although the former symptoms were improved or gone.

In our group, no difference in the mean HRQoL between surgically and conservatively treated patients was seen. This might be because the patients who responded to conservative treatment did not undergo surgery and therefore had a similar outcome. Furthermore, there was a much lower response rate to the questionnaire among the conservatively treated patients (38.5% versus 80.2% in the surgically treated group). Due to the low number of non-surgically treated patients, no conclusion concerning the comparison of surgically and non-surgically treated patients can be drawn from our results.

## Limitations

Due to its design, this study had several limitations. First, this was a single-center analysis. However, this is one of the first studies on the social impact and HRQoL in patients with SIH. Second, the questionnaire was sent to the patients on average 2 years after treatment and not prospectively at well-defined time points, which could have led to a recall bias for some of the questions. Therefore, we cannot compare the HRQoL before and after treatment. Furthermore, data collection with a questionnaire was not anonymous, so there might have been a selection bias in answering. Prospective studies and databases would be needed to exclude this bias. Third, the HRQoL was compared to that of an age- and sex-standardized sample of the general Finnish population. Since there are no data for the 15D for the Swiss population, and many patients came from abroad for diagnostic and therapeutic work-up, there was no better option available for comparing our study group to the general population.

## Conclusion

SIH has a notable negative socio-economic impact on the patients’ social life and HRQoL. It causes long periods of incapacity for work and may even lead to early retirement, thus resulting in high economic costs. Additionally, the HRQoL of SIH patients compared to the general population is impaired despite treatment underlining the chronic character of this disease.

## Data Availability

The datasets generated during and/or analyzed during the current study are available from the corresponding author on reasonable request.

## Abbreviations

CSF: Cerebrospinal fluid
HRQoL: Health-related quality of life
MIC: Minimum clinically important change
SD: Standard deviation
SIH: Spontaneous intracranial hypotension
